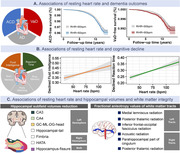# Associations of resting heart rate with incident dementia, cognition, and brain structure: a prospective cohort study of UK biobank

**DOI:** 10.1002/alz.088651

**Published:** 2025-01-09

**Authors:** Deng Yue‐Ting

**Affiliations:** ^1^ Huashan hospital, Fudan University, Shanghai, Shanghai China

## Abstract

**Background:**

Resting heart rate (RHR) has been linked with an increased risk of dementia. However, evidence characterizing the associations of RHR with different dementia subtypes and their underlying mechanisms remains scarce. This study aims to investigate the relationships of RHR with different dementia types, cognitive function, and brain structural abnormalities.

**Method:**

Three hundred thirty‐nine thousand nine hundred one participants with no prior diagnosis of dementia from the UK biobank were analyzed. Cox regression and restricted cubic spline models examined the associations between RHR with all‐cause dementia (ACD) and its major subtypes—Alzheimer’s disease (AD) and vascular dementia (VaD). Logistic regression models assessed the associations of RHR with cognitive function, and linear regression models estimated the associations with hippocampal subfield volume and white matter tract integrity indexed by magnetic resonance imaging data.

**Result:**

During an average of 3148 (± 941.08) days of follow‐up, 4177 individuals were diagnosed with dementia, including 2354 AD and 989 VaD cases. RHR ≥ 80bpm was associated with ACD (HR: 1.18, 95% CI: 1.08–1.28, P < 0.001) and VaD (HR: 1.29, 95% CI: 1.08–1.54, P = 0.005) but not AD in multi‐adjusted models. A 10‐bpm increment of RHR demonstrated non‐linear effects in VaD, consisting of J‐shape relationships. Several heterogeneities were indicated in stratified analysis, in which RHR measures only showed associations with dementia incidents in relatively younger populations (age ≤ 65) and females. Apart from dementia analysis, elevated RHR was associated with worsening performance in fluid intelligence and reaction time of cognitive tasks, decreased hippocampal subfields volume, and poor white matter tract integrity.

**Conclusion:**

RHR is associated with increased risks of ACD and VaD but also presented with few heterogeneities across different sex and age groups. Elevated RHR also appears to have deleterious effects on cognitive function and is distinctively associated with volume reduction in hippocampal subfields and impaired white matter tract integrity.